# A Perspective on the Role of Mitochondrial Biomolecular Condensates (mtBCs) in Neurodegenerative Diseases and Evolutionary Links to Bacterial BCs

**DOI:** 10.3390/ijms26178216

**Published:** 2025-08-24

**Authors:** Matteo Calcagnile, Pietro Alifano, Fabrizio Damiano, Paola Pontieri, Luigi Del Giudice

**Affiliations:** 1 Department of Experimental Medicine, University of Salento, 73100 Lecce, Italy; pietro.alifano@unisalento.it (P.A.); fabrizio.damiano@unisalento.it (F.D.); 2 Sezione di Igiene, Istituto di Bioscienze e BioRisorse-UOS Napoli-CNR c/o Dipartimento di Biologia, 80134 Naples, Italy; paola.pontieri@ibbr.cnr.it

**Keywords:** biomolecular condensates, liquid–liquid phase separation, mitochondria, disordered protein, neurodegenerative diseases, bacteria

## Abstract

Biomolecular condensates (BCs), formed through liquid–liquid phase separation (LLPS), are membraneless compartments that dynamically regulate key cellular processes. Beyond their canonical roles in energy metabolism and apoptosis, Mitochondria harbor distinct BCs, including mitochondrial RNA granules (MRGs), nucleoids, and degradasomes, that coordinate RNA processing, genome maintenance, and protein homeostasis. These structures rely heavily on proteins with intrinsically disordered regions (IDRs), which facilitate the transient and multivalent interactions necessary for LLPS. In this review, we explore the composition and function of mitochondrial BCs and their emerging involvement in neurodegenerative diseases such as Alzheimer’s disease, Parkinson’s disease, Amyotrophic lateral sclerosis, and Huntington’s disease. We provide computational evidence identifying IDR-containing proteins within the mitochondrial proteome and demonstrate their enrichment in BC-related functions. Many of these proteins are also implicated in mitochondrial stress responses, apoptosis, and pathways associated with neurodegeneration. Moreover, the evolutionary conservation of phase-separating proteins from bacteria to mitochondria underscores the ancient origin of LLPS-mediated compartmentalization. Comparative analysis reveals functional parallels between mitochondrial and prokaryotic IDPs, supporting the use of bacterial models to study mitochondrial condensates. Overall, this review underscores the critical role of mitochondrial BCs in health and disease and highlights the potential of targeting LLPS mechanisms in the development of therapeutic strategies.

## 1. Introduction

Liquid–liquid phase separation (LLPS) is a thermodynamic process where a homogeneous aqueous solution spontaneously separates into two immiscible liquid phases: a dense phase enriched in specific biomolecules and a dilute phase [1,2,3]. In the cellular context, LLPS occurs when intermolecular interactions between macromolecules, including proteins and nucleic acids, become more energetically favorable than those with surrounding solvent, triggering the formation of biomolecular condensates (BCs) [1,2,3]. Key factors influencing this process include macromolecular concentration, temperature, ionic strength, pH, the balance of hydrophilic and hydrophobic interactions, and more [4,5,6]. Often, interactions between macromolecules include weak and transient interactions driven by electrostatic forces (e.g., charge–charge interactions), π-π stacking, and hydrophobic interactions [7] that typically occur between intrinsically disordered regions (IDRs) of proteins and nucleic acids determine LLPS [7].

LLPS is of fundamental importance for medicine and biology by governing the formation BCs and membraneless organelles (MLOs) [8,9,10], like nucleoli, stress granules, and P granules, which compartmentalize cellular processes without lipid membranes [10,11,12]. LLPS and BCs contribute to the spatial and temporal organization of key cellular processes, including heterochromatin assembly, transcriptional and translational regulation, stress response, and signal transduction [13].

IDRs are protein domains that lack a stable, well-defined three-dimensional structure under physiological conditions and display high conformational flexibility [14]. In contrast, most proteins contain rigid, folded domains stabilized by hydrogen bonds, hydrophobic interactions, and other non-covalent forces. IDRs, instead, exist as ensembles of rapidly interconverting conformations, resulting in structural heterogeneity [14]. Their length can vary significantly, from short flexible linkers to extensive disordered segments. Typically, IDRs undergo structural stabilization upon interaction with binding partners, enabling the formation of complex macromolecular networks [15].

The phenomenon of BCs formation by LLPS is well known, especially in eukaryotes, where BCs are formed in the cytoplasm and nucleus [16] and in some cases have been identified as MLOs. However, BCs also exist in other organisms, including bacteria and archaea [16]. Furthermore, BCs have also been detected and described in organelles, such as mitochondria and chloroplast [16].

Mitochondria host a variety of BCs that compartmentalize key processes involved in RNA metabolism and genome maintenance. Among these, mitochondrial RNA granules (MRGs) serve as hubs for newly synthesized RNA, processing enzymes, and mitoribosome assembly factors, and are typically associated with the inner mitochondrial membrane. Mitochondrial nucleoids, composed of mitochondrial DNA and architectural proteins like TFAM, are essential for genome organization and replication, and can undergo stress-induced clustering linked to mitochondrial dysfunction. Additional condensates, such as RNA degradasomes, are emerging as potential LLPS structures involved in RNA surveillance and turnover, although they remain less characterized [17,18,19,20].

Chloroplasts also harbor BCs, including chloroplast stress granules (cpSGs), which form in response to various stress conditions, particularly oxidative and heat stress. These granules are enriched in RNA-binding proteins, small ribosomal subunit components, and untranslated mRNAs, and are believed to assemble via LLPS. Their composition suggests a protective role in modulating plastidial gene expression during stress by transiently sequestering translation-related components and transcripts [18,21].

Several parallels exist between molecular condensates in prokaryotes and those in eukaryotes, including within organelles. In bacteria, molecular condensates also form through the interaction between RNA and proteins. For instance, bacterial RNA polymerase forms clusters that function as molecular condensates [22]. Additional proteins involved in RNA processing and degradation, such as RNase E, Hfq, and Rho, also participate in the formation of these condensates [23,24,25,26]. Similarly, proteins involved in chromosome organization, including Dps, HU, and SSB, form molecular condensates [23,27,28,29]. Finally, as observed in eukaryotic cells, bacteria can form granules in response to stress, such as aggresomes and polyphosphate granules [23,30,31]. As demonstrated in eukaryotes, the formation of BCs in prokaryotes is also influenced by ATP levels, further highlighting the evolutionary conservation of certain aspects of condensate biology [32].

The phenomenon of LLPS and the formation of BCs has gained increasing attention in recent years. LLPS plays a key role in various human pathologies, including cancer, neurodegenerative disorders, genetic diseases, and viral infections [2]. Importantly, mitochondria dysfunction has also emerged as a key player in many diseases, particularly in cancer and neurodegeneration. It is a hallmark of several neurodegenerative disorders, including amyotrophic lateral sclerosis (ALS), frontotemporal dementia (FTD), Huntington’s disease (HD), tauopathies, Alzheimer’s disease (AD), and Parkinson’s disease (PD) [33,34,35,36,37], and contributes to the loss of neuronal function and structure. In neurodegeneration, genetic mutations and cellular stress can affect the molecules forming BCs, altering their physical properties and behavior. This often leads to a transition from dynamic, liquid-like condensates to more solid, gel-like aggregates. Such changes promote the formation of pathological protein inclusions seen in ALS, FTD, HD, tauopathies, AD, and PD [38,39,40,41].

This review provides an overview of the role of mitochondria and cytoplasmic BC in neurodegenerative diseases. It also discusses the still understudied functions of mitochondrial BCs in neurodegenerative diseases. Finally, the review highlights the parallels between bacteria and mitochondria, emphasizing the value of prokaryotes as models for studying mitochondrial BCs (mtBCs).

## 2. Pathological Implications of LLPS and BCs

In recent years, the phenomenon of LLPS has attracted growing interest due to its involvement in the pathogenesis of several diseases as a consequence of altered BC formation. Aberrant condensates can lead to altered function by either gaining anomalous functions through the accumulation of pathogenic protein aggregates, or by losing physiological functions by disrupting essential cellular processes. For example, neuronal BCs involved in axonal transport and synaptic plasticity may become dysfunctional, leading to impaired transport, synaptic failure, and excitotoxicity [38,42]. Given the importance of BCs in diseases, particularly neurodegenerative ones, several approaches have been proposed to modulate BC formation [43,44,45,46,47]. Furthermore, there has recently been growing interest in the regulation of BC formation and its role in neurodegenerative diseases [45,48,49,50]. Notably, BCs are involved in splicing processes and, conversely, splicing can regulate the formation of condensates [51]. BCs are also involved in neurodegenerative diseases caused by prions [52,53]. Proteolysis has been shown to control the initiation of aberrant phase transitions of BCs into aggregates in a circuit involving the protein Clusterin as a quality checkpoint to prevent the formation and propagation of proteins with prion-like conformations [54].

The dynamic nature of BCs makes them highly vulnerable to various biological factors, including alterations caused by genetic mutations, aging, and cellular stress [38,55]. For example, mutations in IDRs can trigger aberrant intermolecular interactions critical for pathological BC formation, while aging is associated with the reduced efficiency of protein quality control systems [38]. Several proteins participating in BC formation are directly implicated in neurodegenerative diseases, such as ALS, FTD, AD, and PD. Key examples include TDP-43 and FUS (ALS/FTD) [56], tau (AD) [57], α-synuclein (PD) [58], and hnRNPA1 (ALS) [59,60,61]. The formation of these defective BCs has functional consequences that contribute to neurodegeneration by compromising the normal function of these proteins in axonal transport and synaptic signalling.

Interestingly, BCs can also play protective roles. Some studies indicate that, under certain conditions, condensates can sequester aggregation-prone proteins such as α-synuclein (linked to PD), stabilizing them and suppressing their aggregation. This suggests a complex dual role for condensates in neurodegeneration, both facilitating and potentially mitigating protein aggregation depending on the context [62].

### 2.1. TDP-43 and FUS in ALS and FTD

TDP-43 (TAR DNA-binding protein 43) is a ubiquitously expressed RNA/DNA-binding protein that plays a central role in RNA metabolism, particularly within neuronal cells. It is primarily located in the nucleus under normal conditions but is capable of shuttling between the nucleus and cytoplasm. Structurally, TDP-43 contains two RNA recognition motifs (RRMs) that allow it to bind single-stranded RNA and DNA, with a particular affinity for UG-rich sequences. The N-terminal domain contributes to protein stability and dimerization, while the C-terminal region, intrinsically disordered and rich in glycine, glutamine, and asparagine residues, is an IDR with prion-like features that promotes pathological aggregation [63]. Functionally, TDP-43 regulates several aspects of RNA processing, including alternative splicing, mRNA transport, stability, and translation [64].

TDP-43 aggregation of TDP-43 is a key pathological hallmark of ALS and FTD [65,66]. Aggregation of TDP-43 results in the formation of amorphous inclusions in cells. Most ALS- and FTD-linked mutations are located in this domain and increase its aggregation propensity and cellular toxicity. Experimental models, ranging from in vitro assays with purified protein to yeast and mammalian cell cultures, confirm the involvement of the C-terminal domain in protein aggregation [63,65].

Therapeutic approaches targeting mutated TDP-43 include allele-specific siRNAs. For example, siM10 is siRNA that selectively silences the expression of TDP-43 allele with the p.G376D mutation in ALS fibroblasts, reducing cytoplasmic TDP-43 inclusions, and restoring of TDP-43 nuclear localization, oxidative redox, and cell viability [67]. Similarly, in iPSC-derived neurons from ALS patients, the use of allele-specific siRNAs targeting the M337V mutation show efficacy in reducing cytosolic TDP-43 aggregates [68]. Furthermore, computational approaches have been proposed to identify small molecules that interfere with BC formation by TDP-43 without causing loss of protein functionality [69].

In FTD, TDP-43 undergoes cleavage, hyperphosphorylation, and ubiquitination, leading to its mislocalization from the nucleus to the cytoplasm and subsequent aggregation [66,70,71]. Under stress conditions, TDP-43 accumulates in cytoplasmic stress granules as aggregates, that may propagate between cells via exosomes or tunnelling nanotubes, supporting a prion-like diffusion in the brain [66,70,71,72,73]. These aggregates, with ubiquitin-positive and tau-negative inclusions, define a class of neurodegenerative diseases known as TDP-43 proteinopathies [71].

FUS (Fused in Sarcoma) is another ubiquitously expressed RNA/DNA-binding protein belonging to the FET family, comprising around 526 amino acids, which plays a central role in the pathogenesis of both FTD and ALS. FUS is predominantly intrinsically disordered, with a well-structured central domain. Its N-terminal region is rich in glutamine, glycine, serine, and tyrosine (QGSY), followed by three arginine–glycine–glycine (RGG) repeats. The central region contains an RNA recognition motif (RRM) and a Cys2–Cys2 zinc-finger motif, while a nuclear localization signal (NLS) resides at the C-terminus. FUS binds RNA and DNA through its RGG–ZnF–RGG and RRM domains, with structural studies revealing a bipartite mechanism: the zinc finger engages GGUG motifs in RNA, whereas the RRM makes more general shape-based contacts, aided by a distinctive “KK” positively charged loop. In the nucleus, FUS regulates critical aspects of RNA metabolism, including transcription, alternative splicing, mRNA stability, microRNA processing, and even DNA repair via its recruitment to damage sites. In neurons, it also supports RNA transport and localized translation, contributing to dendritic spine maturation [74].

Under physiological conditions, FUS forms reversible, highly dynamic nuclear liquid condensates, facilitating RNA processing [75,76,77]. Mutations, especially in glycine/arginine-rich regions, disrupt its phase behavior, promoting cytoplasmic aggregation and reducing condensate fluidity [75,76,78]. These cytosolic aggregates are a hallmark pathological feature of both ALS and FTD [79].

### 2.2. Tau and α-Synuclein in AD and PD

Tau is a microtubule-associated protein with an IDR encoded by the MAPT gene predominantly expressed in neurons, where it plays a key role in stabilizing the microtubule cytoskeleton. Its sequence comprises several functionally distinct regions, including an acidic N-terminal projection domain, a central proline-rich region, and a C-terminal microtubule-binding region that contains three or four imperfect repeat motifs (R1–R4 repeats), depending on the isoform. FUS can undergo LLPS forming cytoplasmic and nuclear BCs that participate in physiological functions including chromatin compaction, DNA protection, and microtubule assembly [80,81,82].

Under pathological conditions, tau condensates can become solid aggregates, forming neurofibrillary tangles, a pathological hallmark of AD [82]. Phase separation is promoted by interactions with polyanions (e.g., RNA, DNA) and modulated by post-translational modifications (PTMs) such as phosphorylation and ubiquitination [80,81,83]. Tau nanosized-condensates are located in synapses regulating the organization of synaptic vesicles and may trigger toxic amyloid fibril formation [82].

α-Synuclein (α-syn) is a small, 140-amino acid protein highly expressed in the central nervous system, especially at presynaptic terminals, where it is thought to play a role in the regulation of synaptic vesicle trafficking, recycling, and neurotransmitter release. Structurally, its sequence is composed of three major regions: an amphipathic N-terminal domain that binds lipid membranes and adopts α-helical conformations upon membrane interaction; a central hydrophobic region known as the NAC (non-amyloid-β component), which is critical for aggregation; and a highly acidic, proline-rich C-terminal domain, which remains disordered and modulates interactions with other proteins and metals [84]. α-syn is the major component of Lewy bodies in PD, and can undergo LLPS, especially under conditions of molecular crowding or in the presence of mutations/PTMs [81,85].

In vitro, spontaneous phase separation requires higher concentrations of α-synuclein than tau and is facilitated by molecular factors such as PEG [85]. Within these condensates, α-synuclein aggregates more efficiently, suggesting that LLPS accelerates amyloidogenesis in PD patients [40,82,86]. Moreover, tau and α-synuclein may synergize in aggregate formation, particularly in PD with dementia [87]. Further, α-synuclein can partition into tau/RNA droplets, especially in its full-length form, and this interaction is modulated by post-translational modifications such as phosphorylation. This co-localization within condensates may contribute to the synergistic progression of neurodegenerative pathologies [82,87].

## 3. The Role of Mitochondria in Neurodegenerative Diseases

The human mitochondrial genome is a circular DNA molecule consisting of 16,569 base pairs and encodes 37 genes: 13 proteins essential for the mitochondrial respiratory chain and oxidative phosphorylation (OXPHOS), two ribosomal RNAs (12S rRNA and 16S rRNA), and 22 transfer RNAs (tRNAs) [33,37,88,89,90]. Over the past four decades, extensive research has highlighted the critical role of mitochondria in both normal brain function and the development of mitochondrial-related diseases affecting the nervous system [91]. Collectively, “mitochondrial disease” refers to a diverse group of disorders caused by defects in the formation and/or function of oxidative OXPHOS proteins [92]. These pathologies arise from inherited or spontaneous mutations in mitochondrial DNA (mtDNA) or nuclear DNA (nDNA), which impair the expression or mitochondrial proteins or RNAs [89,92,93]. Given the central role of mitochondria in ATP production, calcium homeostasis, redox balance, and apoptotic signaling [94,95], mitochondrial dysfunction significantly contributes to numerous pathological conditions, including aging [96,97], cancer, metabolic syndromes, and particularly neurodegenerative diseases such as AD and PD [98,99]. In recent years, substantial efforts have clarified some of the underlying mechanisms of neurodegeneration, particularly in the two most prevalent neurodegenerative diseases, AD and PD.

### 3.1. Role of Mitochondria Dysfunction in Alzheimer’s Disease

Mitochondria are increasingly recognized as central players in the development and progression of AD. Dysfunction in these organelles has been closely linked to impaired energy metabolism, elevated oxidative stress, and widespread neuronal damage. Current research suggests that mitochondrial impairment may not be merely a consequence of AD pathology but rather an early and initiating event in the neurodegenerative process.

Under normal conditions, mitochondria produce ATP through OXPHOS, providing neurons with the energy required to synaptic activity, ionic gradients and overall cell viability [100,101,102]. In AD, OXPHOS is significantly impaired, leading to reduced ATP production and subsequent neuronal dysfunction. These energy deficits are particularly detrimental in metabolically demanding brain regions such as the hippocampus and cortex [100,101,102]. Another critical hallmark of mitochondrial dysfunction in AD is the overproduction of reactive oxygen species (ROS) [101,103]. While mitochondria normally generate ROS under physiological conditions as a byproduct of respiration, in AD this production becomes excessive, leading to oxidative damage to mtDNA, lipids, and proteins. This oxidative stress exacerbates neuronal injury and promotes neurodegeneration. Moreover, amyloid-beta (Aβ) accumulation has been shown to further elevate ROS production, establishing a harmful vicious cycle that amplifies mitochondrial and neuronal damage [104,105].

A crucial aspect of mitochondrial homeostasis involves the balance between mitochondrial fusion and fission, collectively known as mitochondrial dynamics. These processes are mediated by specific proteins: mitofusins 1 and 2 (MFN1/2) and OPA1 regulate fusion, promoting mitochondrial elongation and content mixing, while dynamin-related protein 1 (DRP1) drives fission, enabling mitochondrial division and quality control [106,107]. In AD, this balance is disrupted, favoring excessive fission over fusion. This imbalance leads to mitochondrial fragmentation, impaired transport along neuronal axons, and insufficient energy delivery to synaptic terminals [108,109]. Moreover, altered dynamics compromise mitophagy—the selective removal of damaged mitochondria—resulting in their accumulation and further aggravating intracellular stress [108,109]. Emerging evidence indicates that mitochondrial abnormalities occur early in AD, and can be detected before the formation of classical pathological hallmarks such as Aβ plaques and tau neurofibrillary tangles, suggesting that mitochondrial dysfunction may initiate and possibly trigger the neurodegenerative cascade [110,111].

In this contest, Aβ not only localizes within mitochondria but also disrupt calcium signalling between mitochondria and the endoplasmic reticulum (ER), ultimately disturbing cellular metabolic regulation [112].

Additionally, hyperphosphorylated tau impairs axonal transport by destabilizing microtubules and disrupting the interaction between motor proteins (such as kinesin-1) and mitochondria, leading to significant reductions in anterograde mitochondrial movement toward synapses and consequent energy deficits and oxidative stress in neuronal processes [113]. Additionally, tau can accumulate within mitochondria and has been shown to interact with key components of the electron transport chain, exacerbating defects in oxidative phosphorylation and increasing reactive oxygen species production [113]. These disruptions not only compromise mitochondrial distribution and function but also potentiate synaptic failure and neuronal loss. Furthermore, tau-mediated transport deficits sensitize neurons to amyloid-beta toxicity: reducing tau levels in AD models alleviates Aβ-induced impairments in mitochondrial motility, highlighting a synergistic interplay between tau and Aβ in mitochondrial and synaptic pathology [113].

### 3.2. Role of Mitochondria Dysfunction in Parkinson’s Disease

PD is a progressive neurodegenerative disorder primarily characterized by the selective loss of dopaminergic neurons in the substantia nigra pars compacta, which leads to hallmark motor symptoms such as bradykinesia, rigidity, and tremor. As previously discussed, a growing body of evidence suggests that mitochondrial dysfunction plays a central role in PD pathogenesis by influencing neuronal survival through multiple mechanisms, including impaired bioenergetics, increased oxidative stress, and failure of mitochondrial quality control systems [114,115,116,117]. Post-mortem analyses have revealed oxidative modifications of proteins and down-regulation of ubiquitin carboxyl-terminal hydrolase L1 (UCH-L1) in the brains of both PD and AD patients [118].

Among mitochondrial defects, mitochondrial complex I (MCI) activity is found to be deficient in PD, particularly in the substantia nigra, the region where dopaminergic neuronal loss is most pronounced [119]. Importantly, MCI dysfunction alone has been shown to be sufficient to induce progressive parkinsonism in experimental models, suggesting it may act as a primary causative factor rather than merely a downstream consequence of disease progression [120].

Mechanistically, MCI dysfunction contributes to dopaminergic neuron death through activation of p53-mediated Bax transcription and JNK/Bim-dependent Bax translocation. Bax (Bcl-2-associated X protein) is a pro-apoptotic member of the Bcl-2 family that promotes mitochondrial outer membrane permeabilization, leading to cytochrome c release and activation of caspases. Notably, blocking either of these mechanisms has been shown to significantly reduce neurodegeneration, identifying potential therapeutic targets [121].

MCI deficiency is not limited to the substantia nigra, but it has also been observed in other brain regions and peripheral tissues such as skeletal muscle and platelets, suggesting a systemic mitochondrial dysfunction in a subset of PD patients [122,123]. Based on distribution and severity of MCI deficits, idiopathic PD patients have been stratified into two subtypes, CI-PD, characterized by widespread mitochondrial dysfunction and predominantly non-tremor symptoms, and nCI-PD, which presents more localized dysfunction and predominantly tremor-dominant features [124]. This stratification reveals distinct molecular and clinical profiles, aiding in targeted research and therapy [124].

Consistent with findings in AD, disruptions in mitochondrial dynamics and quality control mechanisms, especially mitophagy, have also been identified as central contributors to the pathogenesis of familial PD. Dysregulation of these processes leads to mitochondrial fragmentation or elongation, impairing mitochondrial function and neuronal health. Notably, in familial PD, genes such as PINK1 and Parkin control mitochondrial quality through mitophagy, the selective autophagic removal of damaged mitochondria [125,126,127,128]. Mutations in these genes disrupt mitophagy, leading to the accumulation of dysfunctional mitochondria, increased oxidative stress, and progressive neuronal degeneration.

Other genes involved in mitochondrial maintenance include DJ-1 and HTRA2. DJ-1 acts as a redox-sensitive chaperone and oxidative stress sensor. It participates in preserving mitochondrial function by activating antioxidant responses through the Nrf2 pathway. Mutations in DJ-1, such as L166P, impair its antioxidant activity, leading to increased ROS and mitochondrial dysfunction [129]. HTRA2/Omi is a mitochondrial serine protease involved in protein quality control. Intriguingly, HTRA2, helps degrade misfolded proteins within mitochondria and can trigger apoptosis when mitochondrial stress is severe. Moreover, HTRA2 has been shown to cleave mutant DJ-1 (L166P), linking the two proteins in a shared pathway of mitochondrial regulation and neuroprotection [129,130]. Also, a synergistic reduction in mitochondrial protein quality control, including PINK1, Parkin, and mitochondrial chaperones was observed in dopaminergic neurons from PD patients using imaging mass cytometry (IMC) [117].

Mitochondrial dysfunction in PD is further exacerbated by the pathological accumulation of α-Synuclein, whose altered location into mitochondria contributes interfering with their morphology, axonal trafficking, and bioenergetic function. This interaction disrupts mitochondrial dynamics and impairs synaptic energy supply, thereby compounding neuronal vulnerability [131,132,133]. These findings highlight the multifaceted impact of mitochondrial impairment in PD, which arises from a convergence of genetic, proteostatic, and metabolic insults affecting neuronal resilience and survival.

## 4. Liquid–Liquid Phase Separation (LLPS) in Mitochondria

Recent studies have revealed that core components of the mitochondrial transcription machinery, including mtDNA, transcription factors, and RNA polymerase, can self-assemble into multiphasic, viscoelastic condensates both in vitro and in vivo. These findings suggest that mitochondrial BCs provide a dynamic, membraneless framework for coordinating multiple aspects of mitochondrial gene expression and homeostasis.

Within mitochondria, several types of mtBCs have been identified, each playing a specific role in RNA metabolism, genome organization, and protein homeostasis. These include mitochondrial RNA granules (MRGs), mitochondrial nucleoid, and mitochondrial RNA degradasomes.

MRGs are subcompartments enriched in newly synthesized RNA, RNA processing proteins, and mitoribosome assembly factors [17]. These granules typically associate with the inner mitochondrial membrane, and such, interaction has been linked to mitochondrial remodeling, including morphological changes and cristae organization [17].

Another prominent mtBC is the mitochondrial nucleoid, a structure composed of mtDNA, transcription factors, including TFAM, and additional proteins essential for mitochondrial gene expression and maintenance [19]. Nucleoids are now recognized as phase-separated condensates whose structural integrity is essential for mitochondrial genome stability. Under oxidative stress, nucleoids undergo clustering and fusion, phenomena associated with transcriptional repression, altered mitochondrial distribution, and impaired respiratory function [20].

In addition to MRGs and nucleoids, mitochondria also harbor RNA degradasomes, and discrete foci that co-localize with mitochondrial transcripts and nucleoids. These structures are thought to be involved in RNA surveillance and turnover, contributing to mitochondrial transcriptome quality control [134]. Although less extensively characterized, RNA degradasomes are hypothesizid to form via LLPS, similarly to their bacterial counterparts, and may play a conserved role in RNA degradation [135].

Recent findings have shown that mtBCs actively modulate mitochondrial transcription. Intriguingly, transcription occurring within these condensates is significantly less efficient than in homogeneous solution [136]. This apparent attenuation is not merely inhibitory but functionally relevant, likely contributing to the fine-tuning of mitochondrial gene expression. It suggests that the physicochemical properties of the condensate environment, such as macromolecular crowding, viscosity, and molecular partitioning, can influence the accessibility and activity of the transcriptional machinery [136].

Furthermore, RNA synthesis exert a feedback effect on condensate structure: the accumulation of nascent RNA within the condensate promotes the formation of vesicle-like, dynamically arrested substructures, profoundly altering the internal organization and phase behavior of the compartment [136]. This reveals a bidirectional relationship between structure and function in these systems: while the condensate environment shapes transcriptional dynamics, in turn, RNA production remodel the structural and rheological properties of the condensate [136].

In living cells, mitochondrial nucleoids remain condensed and droplet-like, with diameters around 100 nm in diameter [137]. As transcription proceeds, newly synthesized mitochondrial RNAs are radially relocalized to separate RNA-processing granules, thereby establishing themselves distinct BCs [136]. This spatial compartmentalization ensures local equilibrium and facilitates efficient gene expression within the mitochondrial network [136].

Together, these findings highlight how LLPS condensates function as adaptive hubs that dynamically regulate gene expression, stress responses, and protein/RNA turnover in mitochondria. By organizing transcriptional and post-transcriptional activities within specialized, membraneless compartments, mitochondria exemplify how LLPS contributes to cellular organization at multiple levels, bridging biochemical compartmentalization with physiological resilience.

### Correlation Between mtBCs and Neurodegenerative Diseases

Several proteins have been investigated for their capacity to form MRGs, and some of these have been associated with human diseases (Table 1). The prevailing hypothesis is that mtBCs serve as hubs for the processing and maturation of mitochondrial RNAs, the assembly of mitoribosomes, and the synthesis of mitochondria-encoded proteins.

Among the proteins annotated as mtBCs proteins in the CD-CODE database [138], some are also implicated in some pathologies and have been previously discussed in literature for their importance in the contex of neurodegenerative diseases [33,37,139,140] (Table 1). These include some mitoribosomal proteins (MRPS31, MRPS7, MRPS9, and MRPS15), mitoribosome-associated helicases (DHX30 and DDX28), mitoribosome-associated methyltransferases (MRM2 and TFB1M), proteins that contribute to the regulation of mitochondrial transcription and ribosome biogenesis (MTERF3, TFB1M, and GTPBP10), and two other particularly interesting proteins: FASTKD2, which coordinates the maturation of mitochondrial mRNAs, and the chaperone ERAL1.

FASTKD2 serves as a central component of MRGs and is required for the processing of polycistronic mitochondrial transcripts into mature RNAs required for mitochondrial translation and oxidative phosphorylation [17,141,142]. It interacts with other RNA-binding proteins such as DHX28 and mitochondrial ribosomal subunits, contributing to ribosome biogenesis [143]. Mutations in the FASTKD2 gene lead to diseases such as Lennox–Gastaut syndrome or a rare form of Mendelian mitochondrial encephalomyopathy [142,144].

DHX30 and DDX28, both RNA helicases, play crucial roles in mitochondrial RNA remodeling and processing, and mitoribosome assembly [141,143,145,146,147,148]. Furthermore, experimental evidence has shown that DHX30 is associated with ribosomes in the embryonic forebrain and is therefore important for brain development [149,150,151,152,153]. Interestingly, DHX30 interacts with FUS, whose mutations are associated with ALS [146]. RNA helicases of the DEAD-box and DEAH-box families play important roles in various RNA-related processes in both prokaryotes and eukaryotes [154]. In bacteria, they are involved in ribosome biogenesis, RNA turnover, and translation initiation, contributing to environmental adaptation through phenotypic changes [155]. In prokaryotes, these helicases also participate in BCs formation and the regulation of LLPS [154,155,156], highlighting an interesting parallel with eukaryotic mitochondria, which are believed to have evolved from proteobacteria, as proposed by the endosymbiotic theory [157,158].

Other components of MRGs include the chaperone ERAL1 and three mitochondrial proteins of the mitochondrial small ribosomal subunit (mtSSU), MRPS7, MRPS9, and MRPS31 [143,147]. ERAL1 acts as an rRNA chaperone that stabilizes 12S rRNA and facilitates the formation of the mtSSU [147,159]. Moreover, ERAL1 positively regulates RNA virus-triggered innate immunity, and mutations of this gene are associated with Perrault syndrome, a disorder characterized by sensorineural deafness and ovarian dysgenesis, often accompanied by neurological deficit [160,161]. In contrast, the mitoribosomal protein MRPS15 was found in the nucleolus, and there is no evidence that it can contribute to MRGs formation [162]. ERAL1 also illustrates an interesting parallel between eukaryotes and prokaryotes. The ERAL1 is homologous to the Era protein identified in *Escherichia coli* (UniProt ID: P06616), where it is involved in ribosome assembly and plays a role in regulating the cell cycle and growth [163,164,165].

Other proteins previously described as mtBCs proteins are MRM2, MTERF3, and TFB1M [147,166,167]. MRM2 is a methyltransferase that modifies mitochondrial rRNA, a critical step in the large ribosomal subunit (mtLSU) [166,168]. MTERF3 is a negative regulator of mammalian mtDNA transcription and mitoribosome biogenesis in mammals and invertebrates [169,170], and the expression level of this gene is correlated with tumor progression and with sporadic cases of developmental delay [171,172]. TFB1M is involved in RNA modifications [173] and is a crucial factor involved in type 2 diabetes [174,175] and, along with MTERF3, in tumor progression [176].

## 5. Intrinsically Disordered Regions in Mitochondrial Proteins

As previously mentioned, proteins contain intrinsically disordered regions (IDRs), which are characterized by the absence of stable tertiary structure and often display low sequence complexity, with numerous hydrophobic amino acid residues such as glycine [177,178,179,180,181,182,183]. These features are important for LLPS and enable the formation of BCs together with nucleic acids (RNA and DNA) [177,178,182,183,184].

As discussed previously, IDRs and intrinsically disordered proteins (IDPs) are increasingly recognized as central contributors to the pathogenesis of neurodegenerative diseases. Indeed, this class of proteins includes amyloid-β (Aβ), tau, α-synuclein, TDP-43, and huntingtin, and exhibit a high propensity for misfolding and aggregation, which underpins the formation of pathological inclusions such as neurofibrillary tangles, Lewy bodies, and cytoplasmic inclusions characteristic of AD, PD, ALS, and HD, respectively [185,186].

The aggregation of IDPs disrupts neuronal homeostasis through multiple mechanisms. First, their structural plasticity and conformational adaptability enable promiscuous interactions with diverse cellular partners, leading to the sequestration of essential proteins and impairment of critical pathways, including proteostasis, signal transduction, and intracellular trafficking [133,187,188,189]. Second, the accumulation of aggregated IDPs overwhelms cellular quality control systems, notably molecular chaperones, the ubiquitin-proteasome system, and autophagic pathways, thereby exacerbating proteotoxic stress and neuronal dysfunction [190].

Machine-learning analyses of proteomic data from neurodegenerative disease models have identified physicochemical properties predictive of IDP inclusion in pathological aggregates, revealing that not all disordered proteins are equally prone to aggregation [191]. Experimental knockdown of selected IDPs in *Caenorhabditis elegans* models of α-synuclein aggregation has resulted in significant reductions in aggregate burden and associated phenotypes, underscoring the functional impact of specific IDPs on disease progression [191]. These findings position IDPs as pivotal mediators of neurodegeneration through their intrinsic disorder-driven aggregation, network perturbations, and phase separation dynamics [192,193,194].

In this review, an independent analysis was conducted by analysing the proteins collected in the MitoCarta3.0 datasets [195]. The analysed dataset contained 2254 protein sequences in FASTA format. Iupred2A was used to study the disorder in these protein sequences [196,197]. IDRs were identified by annotating regions as disordered when more than 10 amino acid residues of the same sequence had a score higher than 0.8. This analysis identified 204 sequences, corresponding to 9% of the initial proteins (Table S1). The initial database contained identifiers (IDs) of repeated genes and therefore 119 proteins were selected (Table S2).

Subsequently, the percentage of occurrence (Relative Abundance, RA) of the 20 amino acids for each protein was calculated (Table S1). Among the selected proteins, some showed high RA (>10%) for hydrophobic amino acids such as glycine, alanine, proline, and valine. BBC3 gene, also known as PUMA, encodes a protein that belongs to the Bcl-2 family and plays a crucial role in inducing apoptosis, and had RA > 10% for alanine, proline, and glycine. Some protein products had an RA > 10% for two nonpolar amino acids: four genes (IVD, ANTKMT, MCL1, and DNLZ) had RA > 10% for alanine and glycine; four genes (BCL2L11, CHCHD10, DLAT, CHCHD2) had RA > 10% for alanine and proline; and three genes (COX6A2, SPHK2, NEU4) had RA > 10% for glycine and proline. Among these genes, MCL1 and BCL2L11 encode proteins belonging to the BCL-2 family, involved in the regulation of apoptosis; DNLZE is involved in the assembly of chaperone-mediated protein complexes; the CHCHD10 gene encodes a protein localized in the mitochondrial intermembrane space, specifically enriched in cristae junctions; COX6A2 encodes the cytochrome c oxidase subunit VIa polypeptide 2; and NEU4 is involved in the regulation of neuronal function. Interestingly, among these genes, CHCHD2 is a significant mitochondrial factor determining alpha-synuclein stability in the etiology of PD.

Other genes show an RA >10% for only one of the three amino acids: 8 genes for glycine (MPV17L2, BAX, MTCH1, G, BAD, TOMM40, PANK2, L2HGDH), 20 for alanine (MGARP, ARMCX2, DNAJC30, BLOC1S1, MICOS13, DCXR, TRMT1, HSPA9, COA3, DELE1, PNKD, DMPK, ATAD3A, TOMM22, BCL2L13, IMMT, TOMM70, ATAD3B, COQ8A, EFHD1), and eight for proline (C6orf136, MAVS, FASTK, MRPS18B, MTX1, DNAJC4, NDUFV3, RTL10).

Interestingly, among the 119 identified genes, 40 are reported to be present in BCs or mtBCs (Table 2), as reported in the CD-CODE dataset [138]. This observation highlights that there is a close link between IDP and BCs, and supports the hypothesis that mtBCs may be central to the development of neurodegenerative diseases. Furthermore, it is interesting to note that many of the genes described in Table 2 are components of presynaptic clusters or postsynaptic densities in humans, as reported in the literature [198] or in CD-CODE dataset [138].

**Figure 1:**
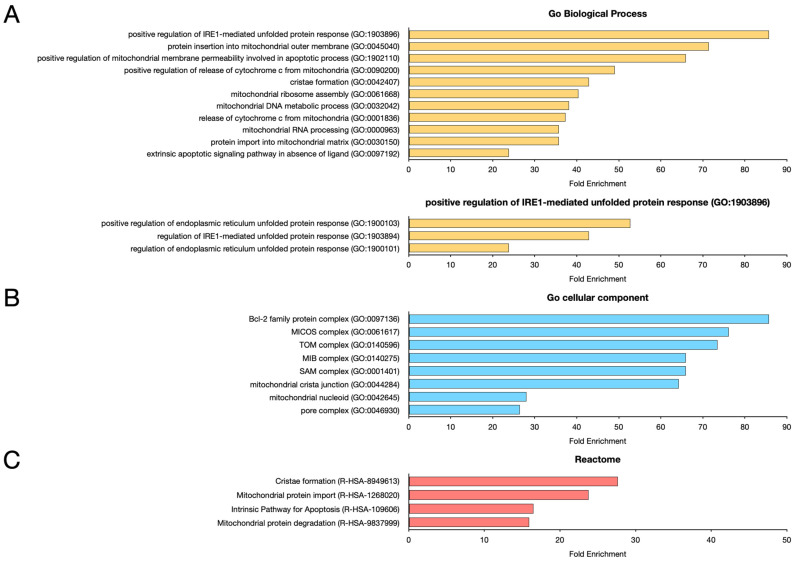
Panther enrichment analysis of mitochondrial gene products showing IDRs. Data were obtained using the Panther tool and Bonferroni correction. (**A**) GO-Biological Process. Only functional classes showing a Fold Enrichment value >20 were reported. (**B**) GO-Cellular Component. Only functional classes showing a Fold Enrichment value >20 were reported. (**C**) Panther Reactome.

To functionally predict which biological processes and cellular functions are associated with the 119 genes presenting protein products containing IDRs, two analyses were performed: a GO-Panther enrichment analysis [199] and an annotation and mapping analysis using the KEGG database [200,201] (Figure 1, Table 3 and Table 4).

As emerges from the Panther analysis (Figure 1C), mitochondrial and endoplasmic reticulum (ER) stress responses are significantly enriched, revealed by the significant enrichment of several surface protein (GO) biological processes. Terms associated with the unfolded protein response, such as IRE1-mediated positive regulation of the unfolded protein response (GO:1903896), regulation of the endoplasmic reticulum unfolded protein response (GO:1900101), and positive regulation of the endoplasmic reticulum unfolded protein response (GO:1900103), suggest an active role of ER stress signaling. In parallel, mitochondria-associated processes, including protein insertion into the mitochondrial outer membrane (GO:0045040), cristae formation (GO:0042407), mitochondrial ribosome assembly (GO:0061668), mitochondrial DNA metabolism (GO:0032042), and mitochondrial RNA processing (GO:0000963), indicate enhanced mitochondrial biogenesis and functional remodeling. Enrichment of protein import into the mitochondrial matrix (GO:0030150) further supports increased mitochondrial activity. Furthermore, the presence of apoptosis-related terms, such as positive regulation of mitochondrial membrane permeability involved in the apoptotic process (GO:1902110), cytochrome c release from mitochondria (GO:0001836), and extrinsic apoptotic signaling pathway in the absence of ligand (GO:0097192), suggest a potential shift toward programmed cell death pathways. These results indicate a coordinated regulation of endoplasmic reticulum and mitochondrial stress responses, potentially reflecting an adaptive mechanism to restore cellular homeostasis or initiate apoptosis under prolonged stress conditions.

Cellular component enrichment analysis (Figure 1B) also revealed that selected mitochondrial genes are membrane-associated, highlighting organelle remodelling and inter-organelle communication. The identification of enriched components such as the Bcl-2 family protein complex (GO:0097136) and the pore complex (GO:0046930) underscores the involvement of apoptotic regulatory mechanisms and nucleocytoplasmic transport in the cellular response. Several mitochondrial substructures, including the MICOS complex (GO:0061617), the TOM complex (GO:0140596), the MIB complex (GO:0140275), and the SAM complex (GO:0001401), were also significantly enriched, reflecting an active regulation of mitochondrial architecture and protein import pathways. The MICOS complex in particular is essential for the maintenance of mitochondrial ridge junctions, which are also represented by the enrichment of the mitochondrial ridge junction terminus (GO:0044284). The presence of the mitochondrial nucleoid (GO:0042645) indicates a potential modulation of mitochondrial DNA organization and transcription. Together, these data suggest a tightly coordinated remodelling of mitochondrial structure and function, potentially in response to bioenergetic demands or stress signals involving both apoptotic and protein homeostasis mechanisms.

Reactome enrichment analysis (Figure 1C) further supports mitochondrial remodelling and apoptotic signaling. Enrichment of cristae formation (R-HSA-8949613) highlights dynamic restructuring of the inner mitochondrial membrane, which is critical for optimizing oxidative phosphorylation and regulating apoptosis sensitivity. Mitochondrial protein import (R-HSA-1268020) and mitochondrial protein degradation (R-HSA-9837999) suggest active turnover and quality control of mitochondrial proteins, indicating tight regulation of mitochondrial proteostasis. Concomitant enrichment of the intrinsic apoptosis pathway (R-HSA-109606) indicates activation of programmed cell death mechanisms, potentially triggered by mitochondrial stress or imbalances in proteostasis. Together, these pathways illustrate a coordinated cellular response involving mitochondrial adaptation, quality control, and apoptotic signalling, consistent with a stress-related or damage-induced phenotype.

KEGG mapping analyses (Table 3 and Table 4) confirmed the association between the selected set of genes and mitochondrial dysfunctions related to neurodegenerative diseases. The results summarized in Table 3 highlight a significant enrichment of genes involved in mitochondrial pathways, apoptosis, and neurodegenerative disease mechanisms. Several key proteins, such as CASP9, BAD, BAX, and BCL2L11 are repeatedly found across multiple pathways, particularly those regulating apoptosis and mitophagy. This suggests a central role for mitochondrial integrity and programmed cell death in the biological system formed by mitochondrial IDPs (mt-IDPs). Notably, pathways related to mitophagy and the intrinsic apoptotic signaling cascade are prominent, indicating active mitochondrial quality control and stress responses. These findings are further supported by the presence of the TOMM and BNIP3 gene families, which mediate mitochondrial outer membrane permeabilization and recruitment of autophagy machinery. The convergence of several of these genes in neurodegenerative disease pathways (AD, PD, ALS, HD, and prion diseases) underscores a shared molecular signature involving mitochondrial dysfunction, oxidative stress, and apoptotic dysregulation. For example, UQCRH, COX6A2, and NDUFV3—components of the electron transport chain—are implicated in all major neurodegenerative pathways listed, suggesting that disruption in mitochondrial respiration is a key driver of pathogenesis.

Mapping was performed using KEGG BRITE (Table 4), a set of hierarchical classification systems that capture the functional hierarchies of biological objects. It highlighted enrichment of genes associated with essential mitochondrial and nuclear processes, particularly those related to the regulation of gene expression, mitochondrial biogenesis, protein folding, and genome maintenance. A major theme emerging from this table is the strong enrichment of mitochondrial biogenesis (38 genes), with several key players involved in mitochondrial DNA replication (POLG, TWNK), protein import (TOMM40, TOMM70, MTX1), translation (MRPL, MRPS subunit, MTERF4), and quality control (LONP1, CHCHD family). The enrichment of ribosomal and RNA biogenesis pathways (e.g., MRPL15, MRPS18B, TRMT1, ERAL1) highlights the importance of mt-IDPs genes for efficient mitochondrial protein synthesis. This complements the enrichment of translation factors and chaperones, such as HSPA9 and CLPB, essential for the correct folding and assembly of mitochondrial proteins. Together, these genes support robust mitochondrial translation and protein quality control mechanisms. Furthermore, the presence of genes involved in DNA repair and replication (RECQL4, LIG3, UNG, OGG1) suggests an active maintenance of mitochondrial and potentially nuclear genome integrity, essential under stress conditions or in cells subjected to oxidative damage. The inclusion of membrane trafficking and mitophagy-related genes (BNIP3, BNIP3L, NBR1) further strengthens the role of dynamic mitochondrial remodeling, including the turnover of damaged organelles, which is closely related to cell survival decisions.

## 6. Evolutionary Conservation of BCs and the Origin of Life

As mentioned previously, BC formation has been verified in eukaryotes and prokaryotes, including archaea and bacteria. Some viral proteins have also been described as BC components [202,203]. Interestingly, viral proteins that form BCs are often nucleocapsid proteins or proteins involved in RNA processing and organization. For example, the RNA-dependent RNA polymerase (RdRp) of the pandemic GII.4 HuNoV virus forms condensates that exhibit all the hallmarks of the LLPS [204]. Similarly, the SARS-CoV-2 nucleocapsid protein forms granules of RNA that are modulated by the virus to maximize replication efficiency [205,206]. These observations suggest that BCs, including the proteins they are made of, are ancestral structures that have been conserved and diversified throughout evolution.

**Figure 2:**
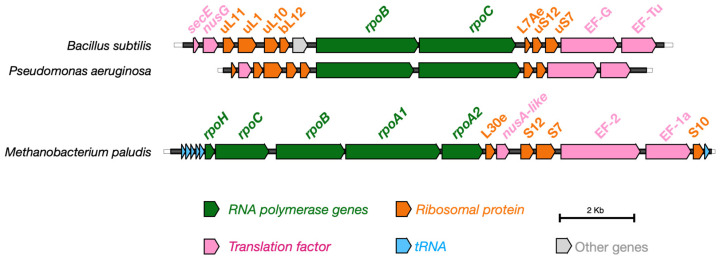
Conservation of the genomic locus containing the genes involved in the synthesis of the RNA polymerase proteins and the formation of ribosomes in bacteria and Archaea.

It is also interesting to note that many of the genes mentioned, such as the bacterial *rpoB* encoding RNA polymerase, the genes encoding ribosomal proteins, and the genes encoding transcription factors such as *nusA*, are vertically inherited [207]. This means that these proteins derive from genes conserved across evolutionary lineages within a species. For example, the NusA and NusG proteins form clusters with RNA polymerase, as recently demonstrated [22]. All these genes mentioned are usually part of a highly conserved genetic locus in prokaryotes, both archaea and bacteria, and show synteny with each other [208,209,210,211]. Figure 2 shows the organization of genes near *rpoB* in bacteria (*Pseudomonas aeruginosa* and *Bacillus subtilis*), archaea (*Methanobacterium paludis*). As can be seen, although the genes are different for different taxonomic groups, the organization of the genetic locus is conserved.

On the other hand, as previously described, some proteins, such as ERAL1, are conserved from bacteria to humans even though ERAL1 is a nuclear-encoded mitochondrial protein [159,163]. This is possible due to the extensive genetic exchange that occurred between the symbiotic bacteria and the host during the process of organelle formation [157]. These additional observations support the hypothesis that the genes involved in BC formation are not only conserved and ancestral, but have also coevolved [212,213]. Supporting this view, it has been proposed that BCs played a fundamental role in the emergence of prebiotic and protobiotic chemistry [213,214].

However, although some proteins that form BCs are preserved, there are important differences between prokaryotes and eukaryotes. On average, Eukaryotic proteins contain about 32% disordered residues, while prokaryotic proteins are only around 12% [215]. This means that an average Eukaryotic Protein has about 145 disordered residues against 32–37 in Prokaryotes [215]. In addition, Eukaryotic IDPs are usually longer than the prokaryotic ones and contain extremely disordered and extensive linker regions that connect more orderly domains [215]. The results obtained from analyzing mitochondrial proteins in this review are consistent with those reported in the literature, as the identified mitochondrial IDPs are small in size and lack extended linker regions [215]. The parallels between mitochondrial and prokaryotic intrinsically disordered proteins (IDPs) highlight the potential of bacteria as a valuable model for studying mtBCs.

## 7. Conclusions

This review has highlighted how LLPS governs the spatial and temporal control of gene expression, particularly within mitochondria, where mtBCs regulate processes such as transcription, RNA processing, and protein homeostasis. Importantly, the dysregulation of these membraneless structures has been increasingly linked to the pathogenesis of neurodegenerative diseases, including AD, PD, ALS, and FTD.

IDRs are present in numerous mitochondrial proteins, suggesting their capacity to form such condensates and respond dynamically to cellular cues. Importantly, the functional enrichment analysis presented here demonstrates a clear relationship of IDR-containing proteins and mtBCs and links these components to neurodegenerative disease pathways. Many of the key proteins implicated in these conditions, including tau, TDP-43, α-synuclein, and FUS, also exhibit phase separation behavior, further reinforcing a shared molecular basis between mitochondrial dysfunction and pathological aggregation.

In addition, evolutionary conservation of LLPS and BC components from bacteria to eukaryotes, including mitochondria, suggests that condensate-based compartmentalization is an ancient and fundamental mechanism of cellular organization. The functional parallels between mitochondrial and prokaryotic IDPs also position bacteria as valuable models for investigating mtBC behavior and dysfunction.

Together, the evidence supports a model in which mtBCs are not only essential for maintaining cellular homeostasis but are also key mediators of stress responses and disease progression. Further understanding of mtBCs and the role of IDRs in their formation may offer new insights into disease mechanisms and therapeutic strategies targeting phase-separated mitochondrial compartments.

## Figures and Tables

**Table 1:** Mitochondrial Ribosomal protein present in BCs according to CD-CODE [138] and associated/candidate disorder.

Gene Name (Uniprot ID)	BCs	Associated/Candidate Disorder	Ref.
DHX30 (Q7L2E3)	mtRNA granule Stress granule P-body PCPD ^1^	Developmental delay Intellectual disability Muscular hypotonia Gait abnormalities Motor and cognitive delay Congenital clasped thumbs Unilateral undescended testicles	[33,139]
DDX28 (Q9NUL7)	mtRNA granule Stress granule	Development and prognosis of colorectal cancer#	[33,139]
ERAL1 (O75616)	mtRNA granule	Perrault syndrome	[33,139]
FASTKD2 (Q9NYY8)	mtRNA granule	Mitochondrial encephalopathy Late age onset autosomal recessive MELAS-like syndrome with optic atrophy mitochondrial encephalomyopathy and hypertrophic cardiomyopathy # * Pancreatic ductal adenocarcinoma prognosis AD # *	[33,139]
MRM2 (Q9UI43)	mtRNA granule	MELAS Non-small cell lung cancer#	[33,139]
MRPS31 (Q92665)	mtRNA granule	Moebius syndrome 1	[33,140]
MRPS7 (Q9Y2R9)	mtRNA granule	Neuritis, with brachial predilection Congenital sensorineural deafness Progressive hepatic and renal failure Lactic acidemia	[33,139,140]
MRPS9 (P82933)	mtRNA granule	Intellectual disability and dysmorphic features	[33,139]
MTERF3 (Q96E29)	mtRNA granule	Multiple cancers *	[33,139]
TFB1M (Q8WVM0)	mtRNA granule	Type 2 diabetes risk *	[33,139]
MRPS15 (P82914)	Nucleolus	Deafness, autosomal dominant nonsyndromic sensorineural 3rd Stuve-Wiedemann syndrome	[33,140]

^1^ PCPD = Presynaptic clusters and postsynaptic densities. # Differential expression. * Copy number variation; gene association, intron variant.

**Table 2:** Genes involved in BCs formation according to the CD-CODE dataset [138] and identified as IDRs-containing genes.

Genes	Presynaptic Clusters & Postsynaptic Densities	Stress Granule	Nucleolus	Centrosome	P-Body	mtRNA Granule	Other
ABCB8	+ ^1^						
AKAP1 ^1^							
ALDH3A2	+ ^2^	+					
ATAD3A		+	+		+		
ATAD3B			+				
BBC3				+			
BCL2L13 ^1^							
CHCHD6	+ ^1^						
DLAT	+ ^1^		+				
ELAC2							PcG body
ERAL1						+	
FASTK		+				+	
GLS	+ ^1^						
GPX4	+ ^1^						
HSPA9	+ ^2^	+	+	+	+		
IDH2	+ ^1^		+				
IMMT	+ ^1^						
LETM1	+ ^1^						
LIG3			+	+			
LONP1	+ ^1^		+				
MTCH1	+ ^1^						
MTHFD1L	+ ^1^		+				
MTX1	+ ^1^		+				
NBR1							P62 cluster
NOA1						+	
NSUN2		+					
OGG1		+		+			Nuclear speckle
OXR1	+ ^1^						
PDHX	+ ^1^						
PNKD	+ ^1^						
PTCD1						+	
PUS1			+				
SCO1		+					
SPHK2				+			
SPHKAP ^1^	+ ^3^						
SPIRE1	+ ^1^						
SUPV3L1						+	
TIMM44			+				
TOMM40			+				
TOMM70	+ ^1^						

^1^ = Protein identified in postsynaptic density in humans [198]. ^2^ = Protein reported in CD-Code [138] as component of postsynaptic density. ^3^ = Protein identified in postsynaptic density only in mice [198].

**Table 3:** Enriched KEGG pathways and associated mt-IDPs coding genes.

KEGG Pathway	Genes
4137 Mitophagy–animal (7) ^1^	*TOMM40 (K11518), BNIP3 (K15464), BNIP3L (K15465), BCL2L13 (K15485), TOMM70 (K17768), MTX1 (K17776), NBR1 (K17987)*
04210 Apoptosis (7) ^1^	*BAD (K02158), BAX (K02159), MCL1 (K02539), CASP9 (K04399), BBC3 (K10132), BAK1 (K14021), BCL2L11 (K16341)*
05200 Pathways in cancer (6) ^1^	*BAD (K02158), BAX (K02159), CASP9 (K04399), BBC3 (K10132), BAK1 (K14021), BCL2L11 (K16341*
05206 MicroRNAs in cancer (4) ^1^	*GLS, GLS2 (K01425), MCL1 (K02539), BAK1 (K14021), BCL2L11 (K16341)*
05210 Colorectal cancer (6) ^1^	*BAD (K02158), BAX (K02159), CASP9 (K04399), BBC3 (K10132), BAK1 (K14021), BCL2L11 (K16341)*
05010 Alzheimer disease (5) ^1^ 05012 Parkinson disease (5) ^1^ 05014 Amyotrophic lateral sclerosis (8) ^1^ 05016 Huntington disease (6) ^1^ 05022 Pathways of neurodegeneration (8) ^1^	*UQCRH (K00416)* ^AD, PD, ALS, HD, ND, PRI^ *COX6A2 (K02266)* ^AD, PD, ALS, HD, ND, PRI^ *NDUFV3 (K03944)* ^AD, PD, ALS, HD, ND, PRI^ *CASP9 (K04399)* ^AD, PD, ALS, HD, ND, PRI^ *BAD (K02158)* ^AD, ALS, ND, PRI^ *BAX (K02159)* ^PD, ALS, HD, ND, PRI^ *TOMM40 (K11518)* ^ALS, ND^ *CHCHD10 (K22759)* ^ALS^ *BBC3 (K10132)* ^HD^ *BAK1 (K14021)* ^PD^

^1^ = N° of genes associated with one pathway. ^AD^ = Alzheimer disease, ^PD^ = Parkinson disease, ^ALS^ = Amyotrophic lateral sclerosis, ^HD^ = Huntington disease, ^ND^ = Pathways of neurodegeneration, ^PRI^ = Prion disease.

**Table 4:** Enriched KEGG Brite classes and associated mt-IDPs coding genes.

KEGG Brite	Genes
ko03000 Transcription factors (1) ^1^	*CHCHD2 (K22758)*
ko03019 Messenger RNA biogenesis (2) ^1^	*HSPA9 (K04043), FASTK (K08290)*
ko03011 Ribosome (3) ^1^	*MRPL15 (K02876), MRPS18B (K16174), MRPL46 (K17427)*
ko03009 Ribosome biogenesis (2) ^1^	*ERAL1 (K03595), NOA1 (K19832)*
ko03016 Transfer RNA biogenesis (6) ^1^	*TRMT1 (K00555), ELAC2 (K00784), TRIT1 (K00791), PUS1 (K06173), NSUN2 (K15335), ATP5MF-PTCD1/PTCD1 (K17710)*
ko03012 Translation factors (1) ^1^	*MTERF4 (K15031)*
ko03110 Chaperones and folding catalysts (5) ^1^	*(CLPB (K03695), HSPA9 (K04043), DNAJC4 (K09524), SPG7 (K09552), DNAJC30 (K19374)*
ko04131 Membrane trafficking (7) ^1^	*SNAP29 (K08509), BNIP3 (K15464), BNIP3L (K15465), NBR1 (K17987), VPS13D (K19527), BLOC1S1 (K20185), PNKD (K23864)*
ko03032 DNA replication proteins (2) ^1^	*(POLG (K02332), RECQL4 (K10730)*
ko03400 DNA repair and recombination proteins (6) ^1^	*POLQ (K02349), UNG (K03648), OGG1 (K03660), RECQL4 (K10730), LIG3 (K10776), EXD2 (K20777)*
ko03029 Mitochondrial biogenesis (38) ^1^	*BAX (K02159), COX11 (K02258), POLG (K02332), ERAL1 (K03595), HSPA9 (K04043), ECSIT (K04405), SCO1 (K07152), ATPAF2 (K07556), LONP1 (K08675), TOMM40 (K11518), BAK1 (K14021), MTERF4 (K15031), BNIP3L (K15465), CHCHD6 (K17564), SUPV3L1 (K17675), TWNK (K17680), ATAD3A, ATAD3B (K17681), ATP5MF-PTCD1,PTCD1 (K17710), TOMM70 (K17768), TOMM22 (K17769), MTX1 (K17776), CHCHD4 (K17782), IMMT (K17785), LETM1 (K17800), TIMM44 (K17804), DNLZ (K17808), MTCH1 (K17885), NBR1 (K17987), COA3 (K18175), NOA1 (K19832), CHCHD10 (K22759), NGRN (K23496), MICOS13 (K24624), OXR1 (K25437), ARMCX2 (K26188), ARMCX6 (K26190), MIGA2 (K27289), PRELID3A (K27966)*

^1^ = N° of genes associated with one class.

## Data Availability

All relevant data are included in the manuscript.

## Supplementary Materials

The following supporting information can be downloaded at: https://www.mdpi.com/article/10.3390/ijms26178216/s1.

## Abbreviations

The following abbreviations are used in this manuscript:

AD	Alzheimer’s disease
PD	Parkinson’s disease
ALS	Amyotrophic Lateral Sclerosis
HD	Huntington’s disease
FTD	Frontotemporal Dementia
LLPS	Liquid–Liquid Phase Separation
BCs	Biomolecular Condensates
mtBCs	Mitochondrial Biomolecular Condensates
MLOs	Membraneless Organelles
ER	Endoplasmic Reticulum
MRGs	Mitochondrial RNA Granules
IDRs	Intrinsically Disordered Regions
IDPs	Intrinsically Disordered Proteins
OXPHOS	Oxidative Phosphorylation
nDNA	Nuclear DNA
mtDNA	Mitochondrial DNA
PTMs	Post-Translational Modifications
RRM	RNA Recognition Motif
NLS	Nuclear Localization Signal
ROS	Reactive Oxygen Species
MFN1/2	Mitofusin 1/2
DRP1	Dynamin-Related Protein 1
siRNA	Small Interfering RNA
TOMM	Translocase of the Outer Mitochondrial Membrane
PINK1	PTEN-Induced Kinase 1
KEGG	Kyoto Encyclopedia of Genes and Genomes
GO	Gene Ontology
